# Evaluation of deep learning-based reconstruction late gadolinium enhancement images for identifying patients with clinically unrecognized myocardial infarction

**DOI:** 10.1186/s12880-024-01308-2

**Published:** 2024-05-31

**Authors:** Xuefang Lu, Weiyin Vivian Liu, Yuchen Yan, Wenbing Yang, Changsheng Liu, Wei Gong, Guangnan Quan, Jiawei Jiang, Lei Yuan, Yunfei Zha

**Affiliations:** 1 Department of Radiology, Renmin Hospital of Wuhan University, No. 238 Jiefang Road, Wuchang District, Wuhan, 430060 China; 2 MR Research, GE Healthcare, Beijing, China; 3 GE Healthcare, Beijing, China; 4 Computer School, Wuhan University, Wuhan, China; 5 Information Center, Renmin Hospital of Wuhan University, Wuhan, China

**Keywords:** Deep learning reconstruction, Diagnostic efficacy, Late gadolinium enhancement, Magnetic resonance imaging, Unrecognized myocardial infarction

## Abstract

**Background:**

The presence of infarction in patients with unrecognized myocardial infarction (UMI) is a critical feature in predicting adverse cardiac events. This study aimed to compare the detection rate of UMI using conventional and deep learning reconstruction (DLR)-based late gadolinium enhancement (LGE_O_ and LGE_DL_, respectively) and evaluate optimal quantification parameters to enhance diagnosis and management of suspected patients with UMI.

**Methods:**

This prospective study included 98 patients (68 men; mean age: 55.8 ± 8.1 years) with suspected UMI treated at our hospital from April 2022 to August 2023. LGE_O_ and LGE_DL_ images were obtained using conventional and commercially available inline DLR algorithms. The myocardial signal-to-noise ratio (SNR), contrast-to-noise ratio (CNR), and percentage of enhanced area (P_area_) employing the signal threshold versus reference mean (STRM) approach, which correlates the signal intensity (SI) within areas of interest with the average SI of normal regions, were analyzed. Analysis was performed using the standard deviation (SD) threshold approach (2SD–5SD) and full width at half maximum (FWHM) method. The diagnostic efficacies based on LGE_DL_ and LGE_O_ images were calculated.

**Results:**

The SNR_DL_ and CNR_DL_ were two times better than the SNR_O_ and CNR_O_, respectively (*P* < 0.05). P_area−DL_ was elevated compared to P_area−O_ using the threshold methods (*P* < 0.05); however, no intergroup difference was found based on the FWHM method (*P* > 0.05). The P_area−DL_ and P_area−O_ also differed except between the 2SD and 3SD and the 4SD/5SD and FWHM methods (*P* < 0.05). The receiver operating characteristic curve analysis revealed that each SD method exhibited good diagnostic efficacy for detecting UMI, with the P_area−DL_ having the best diagnostic efficacy based on the 5SD method (*P* < 0.05). Overall, the LGE_DL_ images had better image quality. Strong diagnostic efficacy for UMI identification was achieved when the STRM was ≥ 4SD and ≥ 3SD for the LGE_DL_ and LGE_O_, respectively.

**Conclusions:**

STRM selection for LGE_DL_ magnetic resonance images helps improve clinical decision-making in patients with UMI. This study underscored the importance of STRM selection for analyzing LGE_DL_ images to enhance diagnostic accuracy and clinical decision-making for patients with UMI, further providing better cardiovascular care.

**Supplementary Information:**

The online version contains supplementary material available at 10.1186/s12880-024-01308-2.

## Background

Myocardial infarction (MI) is diagnosed based on the detection of acute myocardial injury according to cardiac biomarker abnormalities in the context of acute myocardial ischemia [1]. Unrecognized MI (UMI) is a type of MI that has yet to be clinically diagnosed, with the prevalence increasing by 10.0% every decade [2]. Delayed detection due to atypical symptoms can delay treatment, leading to poor prognosis [3]. Failure to achieve reperfusion within a few hours after blood flow cessation may cause myocardial apoptosis in vessel-supplied regions. Therefore, determining the presence or absence of MI and quantifying related variables are crucial in improving the diagnosis, treatment, and prognosis [4, 5].

Cardiac magnetic resonance (CMR) imaging is a promising tool for MI detection because of good tissue contrast and spatial resolution. However, patient compliance is challenging for several reasons, such as the requirement to acquire each high-resolution slice and the need for stable respiration; furthermore, certain conditions, including unstable heartbeat and arrhythmia, can cause motion artifacts on free-breathing scans. As relatively shorter breath-holds are required to acquire more slices, higher-spatial resolution late gadolinium enhancement (LGE) is most frequently utilized in magnetic resonance imaging (MRI) to observe and quantify the degree of myocardial necrosis and microvascular occlusion. Although the enhancement is achieved semi-automatically using post-processing software, the initial sketch of the endocardium, epicardium, enhanced myocardium, and remote normal myocardium relies on the reader’s experience to some extent [6]. Additionally, a previous study reported that LGE could identify only 23 of the 872 participants (2.6%) with UMI [7]. The clinical significance of UMI has been reported using different imaging techniques in diagnosing, refining risk stratification, and guiding clinical decisions for treatments. All underscored the role of CMR in improving the detection accuracy of UMIs, which may affect adverse cardiac outcomes and optimize cardiovascular disease management [8–10]. Therefore, timely and accurate UMI identification and assessment are fundamental for patient stratification and therapeutic planning [4, 5, 11]. In practice, despite many applications of standard deviation (SD) and full width at half maximum (FWHM) techniques, no consensus exists for quantifying scars on LGE images; this challenge persists across different cardiac diseases [12–14]. Obviously, a gap exists in current diagnostic frameworks for analyzing myocardium delayed enhancement.

Deep learning (DL) methods can improve image quality and eliminate intra- and inter-observer variability, enabling more accurate diagnosis and treatment strategies [15, 16] and segmentation for precisely sketched lesions [17–21], among others. However, no DL reconstruction (DLR)-based magnetic resonance (MR) studies have evaluated patients with suspected UMI. Therefore, this study aimed to explore the feasibility and diagnostic performance of DLR-based LGE imaging (LGE_DL_) for patients with UMI compared with that of conventional imaging (LGE_O_) and propose an appropriate signal threshold versus reference mean (STRM) for analyzing LGE_DL_.

## Methods

### Study population

**Fig. 1:**
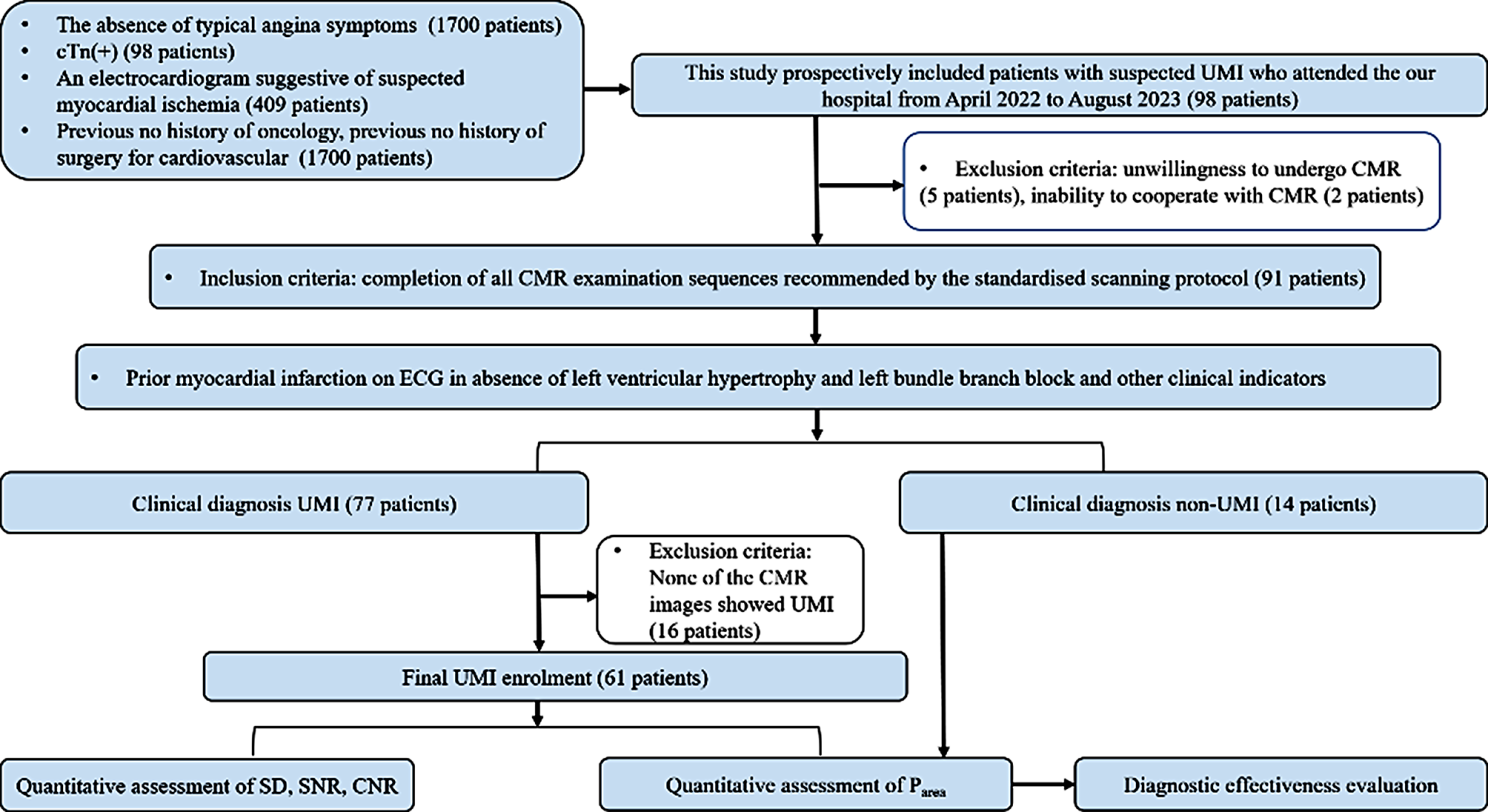
Flowchart of patient enrolment and exclusion. Note: cTn: cardiac troponin; ECG: electrocardiogram; LGE_O_: conventionally constructed late gadolinium enhancement; LGE_DL_: deep learning-based reconstruction late gadolinium enhancement; UMI: unrecognized myocardial infarction; SD: standard deviation; SNR: signal-to-noise ratio; CNR: contrast-to-noise ratio

This study prospectively recruited 98 patients (68 men and 30 women, mean age: 55.8 ± 8.1 years) who presented at our hospital between April 2022 and August 2023 without typical MI symptoms, such as angina pectoris of cardiogenic origin but with suspected UMI after a physical examination. Based on the guidelines of European and American associations and previous reports [1, 7, 22], the inclusion criteria were as follows: (i) the absence of typical angina symptoms; (ii) the presence of elevated or decreased serum cardiac troponin (cTn) levels, with at least one instance of elevation above the upper limit of the normal value (the 99th percentile of the reference value’s upper limit); (iii) prior evidence of MI on electrocardiography in the absence of left ventricular hypertrophy and left bundle branch block; and (iv) no prior history of oncological disease or surgery for cardiovascular diseases. The exclusion criteria were as follows: (i) clinically unstable condition, decompensated heart failure, contraindication to CMR, an estimated glomerular filtration rate ≤ 30 mL/min, and contraindication to the use of gadolinium contrast; and (ii) LGE images that could not be used for clinical diagnosis and objective assessments (Fig. 1).

### CMR examination and image construction

All patients underwent a routine cardiac MRI examination, including a short-axis LGE imaging sequence, on a 3.0-T MRI scanner (Signa Architect, GE Healthcare, Waukesha, WI, USA) at our hospital. A new commercial inline deep-learning-based reconstruction (DLR, brand name: AIR™ Recon DL, DV29.1_R04, GE Healthcare, USA) employs no bias terms and rectified linear unit activations to identify 4.4 million features on directly received image data immediately after scanning on an MR console computer to reduce noise and Gibbs artifacts, and further eliminate intra- and inter-observer differences [13, 16]. The parameters for the LGE sequence were as follows: echo time = 2.7 ms; repetition time = 5.6 ms; flip angle = 25°; field of view = 34 cm × 34 cm; matrix = 260 × 174; slice thickness = 8 mm; slice spacing = 2 mm; receiver bandwidth = 83.33 kHz; views per segment = 24; number of excitations = 1; and theoretical acquisition time = 8 s×nine heart beats. The LGE_O_ and LGE_DL_ were simultaneously generated using conventional inline reconstruction and AIR™ Recon DL algorithms. Fifteen minutes before LGE sequence scanning, a single bolus of 0.1 mmol/kg (0.2 ml/kg) Gadobenate Dimeglumine (Bracco Imaging S.P.A., Milano, Italy) was administered, followed by 20-mL saline flush at a flow rate of 2 ml/s [23]. This dosage was selected based on its efficacy of myocardial enhancement for visualization under the condition of patient safety.

### Assessment of myocardial enhancement area and diagnostic efficacy

Ultimately, data from 61 patients with myocardial enhancement were included in the analysis (43 men [70.5%] and 18 women [29.5%]), with a mean age of 55.9 ± 8.7 years (Fig. 1). The percentage of whole-heart myocardial enhancement area (P_area_) in segments S1–S16 was assessed semi-quantitatively to diagnose cardiovascular disease using Circle Cardiovascular Imaging Inc. (cvi^42^, Circle Cardiovascular Imaging Inc., Calgary, AB, Canada). The delayed enhancement area (i.e., scar size) was subsequently quantified based on threshold methods, which involve adding 2–5 times SD to the mean signal intensity (SI) of the reference myocardium, and the FWHM method, which identifies the half maximum SI at the full width of SI distribution within one region of interest (ROI) in the myocardial tissue. The P_area_ was calculated as the scar size divided by the myocardial volume. Furthermore, the diagnostic efficacy of the P_area_ of LGE_DL_ and LGE_O_ images (P_area−DL_ and P_area−O_, respectively) in differentiating patients with UMI was assessed, with the clinical diagnosis of UMI as the gold standard.

### Theory/calculation

#### CMR image assessment

Qualitative and quantitative imaging evaluations were performed double-blindedly by two radiologists with > 5 years of experience in CMR diagnosis. Moreover, one of the radiologists repeated the assessment 1 month later.

#### Image quality

**Fig. 2:**
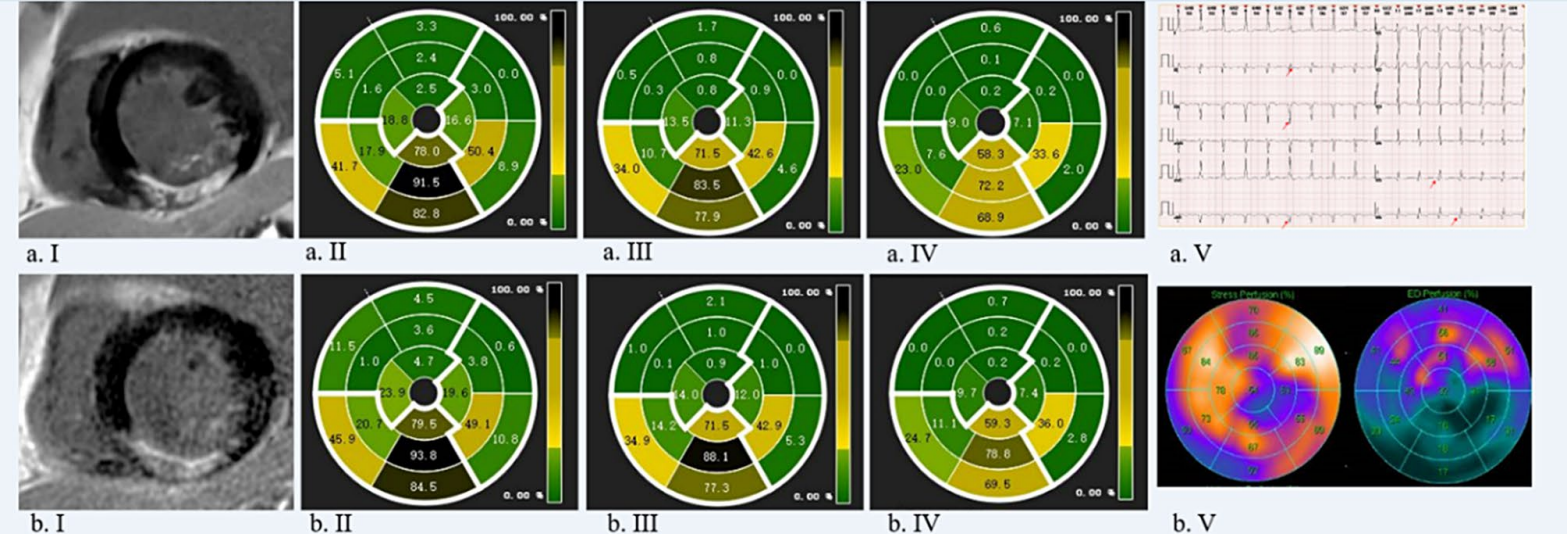
Schematic diagram of P_area_ using accordingly (a.II) and (b.III) 4SD, (b.II) 3SD, (a.III) 5SD, (a.IV) and (b.IV) FWHM methods for (a) LGE_DL_ images, (b) LGE_O_ images, and (a. V) electrocardiogram of a patient with UMI. Figure 2(a) shows clearer, less noisy, more uniform normal myocardial signal and better contrast between the enhancement area and normal myocardium than Fig. 2(b). The patient with UMI underwent stress perfusion myocardium and received an intravenous injection of 20 mCi 99mTc-MIBI. The stress perfusion maps as Fig. 2(b. V) supported our P_area_ maps with clearer myocardium enhancement in the enlarged left ventricle, with the morphological anomaly, relatively light sparsity of 20 mCi 99mTc-MIBI (a radiation tracker, RT) in the middle and basal segments of the anterior wall and the middle segment of the anteroseptal wall, relatively strong sparsity of RTs in the apex, the apical segment of the septal wall, the middle and basal segments of the posteroseptal wall, the apical, middle, and basal segments of the inferior wall, the apical segment of the lateral wall, and the middle and basal segments of posterolateral, and normal perfusion in the remaining myocardium. Note: SD: standard deviation; 2, 3, 4, and 5SD threshold methods: mean P_area_ respectively adding 2, 3, 4, and 5 times of standard deviation of P_area_ as the threshold for myocardial enhancement area; FWHM: full width at half maximum; LGE_DL_: deep learning-based reconstruction late gadolinium enhancement; LGE_O_: conventionally constructed late gadolinium enhancement; UMI: unrecognized myocardial infarction

For the objective evaluation of image quality, ROIs were on LGE_O_ and LGE_DL_ images to determine the SI of the normal myocardium (SI_Myo−O_ and SI_Myo−DL_, respectively) and myocardial delayed enhancement area (SI_MDEA−O_ and SI_MDEA−DL_, respectively), as well as the SD of the background noise at the corner of the images (SD_BG−O_ and SD_BG−DL_, respectively) and the myocardial delayed enhancement area (SD_MDEA−O_ and SD_MDEA−DL_, respectively) (Fig. 2). Additionally, for LGE_O_ and LGE_DL_ images, the myocardial signal-to-noise ratios (SNRs) (SNR_O_ and SNR_DL_, respectively) and contrast-to-noise ratios (CNRs) (CNR_O_ and CNR_DL_, respectively) were calculated [9, 10, 24, 25] using the following formulae:

$$SNR={SI}_{Myo}/{SD}_{BG}$$

$$CNR=|{SI}_{MDEA}-{SI}_{Myo}|/(1.5{SD}_{BG}$$)

The short-axis LGE_O_ and LGE_DL_ images were divided into 16 segments based on the American Heart Association criteria, and the SNR and CNR of each segment were calculated.

### Statistical analysis

All data were statistically analyzed using R-project software (version 4.0.4, http://www.r-project.org). Quantitative data are expressed as either the‾x ± SD or median (interquartile range). All quantitative data were analyzed using either a paired *t*-test or a Wilcoxon signed-rank test depending on the results of the Shapiro–Wilk and Levene’s tests, which were used to assess variance homogeneity and data normality, respectively. To control the false discovery rate, we applied the Benjamini–-Hochberg method for multiple comparison corrections. The intraclass correlation coefficients (ICCs) of the objective quantitative indicators, including the SNR, CNR, SD, and P_area_ for LGE_O_ and LGE_DL_ images (SNR_O_, SNR_DL_, CNR_O_, CNR_DL_, SD_O_, SD_DL_, P_area−O_ and P_area−DL_, respectively) were quantified to assess the degree of intra- and inter-observer agreement. Receiver operating characteristic (ROC) curves for P_area−DL_ and P_area−O_ were constructed using the different threshold methods to determine and compare their diagnostic efficacies for the UMI or non-UMI groups based on the area under the curve (AUC). All statistical significance was set at *P* < 0.05.

## Results

### Patient characteristics

Overall, 77 patients (53 men and 24 women; mean age: 55.6 ± 8.4 years) were diagnosed with UMI based on various clinical indicators, including the cTn level (*n* = 77), imaging features on electrocardiography (*n* = 77), ultrasound cardiography (*n* = 18), computed tomography angiography (*n* = 14), and digital subtraction angiography (*n* = 38), or nuclear medicine test results (*n* = 8). Sixty-one patients (43 men and 18 women; mean age: 55.9 ± 8.7 years) who met the UMI diagnostic criteria were evaluated to assess the distribution of the supplying vessels and the presence of infarction in LGE images. The non-UMI group predominantly exhibited hypertrophic cardiomyopathy (*n* = 10, 71.43%) and left bundle branch block (*n* = 4, 28.57%) (Fig. 1).

### Objective evaluation of image quality

**Fig. 3:**
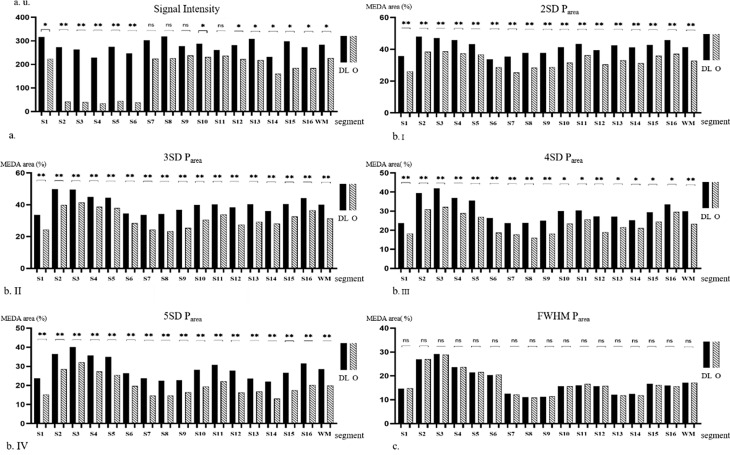
(a) Signal intensity of the left ventricular myocardial on LGE_DL_ and LGE_O_ images. Percentage areas of left ventricular myocardial enhancement in LGE_DL_ and LGE_O_ images using (b. I) 2SD, (b. II) 3SD, (b. III) 4SD, (b. IV) 5SD, and (c) FWHM methods for quantification. Note: SI: signal intensity; WM: whole myocardium; P_area_: percentage of myocardial enhancement area; LGE_DL_: deep learning-based reconstruction late gadolinium enhancement; LGE_O_: conventionally constructed late gadolinium enhancement;; SD: standard deviation; 2, 3, 4, and 5SD threshold methods: mean P_area_ respectively adding 2, 3, 4, and 5 times of standard deviation of P_area_ as the threshold for myocardial enhancement area; FWHM: full width at half maximum; DL, deep learning late gadolinium enhancement; O: original late gadolinium enhancement

The SDs of the normal myocardium, delayed myocardial enhancement areas, and background of the images are presented in Table 1. The SD_DL_ values were lower than the SD_O_ values in all 16 segments, with the S1 segment exhibiting the most significant difference between SD_DL_ and SD_O_ images (31.95 ± 21.82 vs. 45.74 ± 28.29, *P* < 0.05). Overall, the SD_Myo−DL_, SD_MDEA−DL_, and SD_BG−DL_ values of LGE_DL_ images were lower than the respective values of LGE_O_ images, including the SD_Myo−O_ (36.38 ± 19.55 vs. 46.03 ± 18.65, *P* < 0.05), SD_MDEA−O_ (47.39 ± 41.22 vs. 59.77 ± 44.08, *P* < 0.05), and SD_BG−O_ (3.14 ± 2.48 vs. 6.17 ± 4.03, *P* < 0.05). The SNR_DL_ values were higher than the SNR_O_ values in all 16 segments (*P* < 0.001), with the most significant difference observed in the S16 segment (92.44 ± 78.39 vs. 27.39 ± 24.56, respectively; *P* < 0.05). The S1 segment exhibited the highest SNR_DL_ (113.89 ± 98.62), and the S2 segment had the highest SNR_O_ (39.10 ± 41.45). The whole myocardial SNR_DL_ and whole delayed myocardial enhancement CNR_DL_ were significantly elevated compared to the whole myocardial SNR_O_ (99.93 ± 81.42 vs. 33.29 ± 30.89, *P* < 0.05) and whole delayed enhanced myocardium CNR_O_ (123.72 ± 45.00 vs. 60.15 ± 15.52, *P* < 0.05), respectively (Fig. 2a-b–.I, Supplementary Fig. 1a–d.I). The SI_DL_ values were higher than the respective SI_O_ values for all segments (*P* < 0.05) except for S7–S9 and S11. In comparing the SI_Myo−DL_ and SI_Myo−O_ values, the SI_DL_ values were higher than the corresponding SI_O_ values for S1–S6, S10, and S12–S16 (*P* < 0.05). The SI_DL_ values were slightly higher than the corresponding SI_O_ values for S7–S9 or S11; however, the difference was not significant (*P* > 0.05) (Fig. 3a).

**Table 1:** Objective evaluation of image quality for LGE_DL_ and LGE_O_

	SD					SNR			
	LGE _DL_	LGE _O_	t/Z	p		LGE _DL_	LGE _O_	t/Z	p
S1	31.95 ± 21.82	45.74 ± 28.29	5.592	< 0.05		113.89 ± 98.62	33.58 ± 33.36	4.759	< 0.05
S2	36.04 ± 28.27	47.19 ± 30.01	4.522	< 0.05		106.97 ± 103.87	39.10 ± 41.45	3.724	< 0.05
S3	43.34 ± 30.63	52.37 ± 26.63	3.272	< 0.05		98.82 ± 93.38	38.16 ± 41.42	3.667	< 0.05
S4	33.05 ± 20.62	49.28 ± 23.02	5.498	< 0.05		91.24 ± 85.00	32.62 ± 34.86	4.033	< 0.05
S5	39.44 ± 21.07	54.61 ± 24.43	5.111	< 0.05		103.87 ± 98.09	38.78 ± 39.78	3.767	< 0.05
S6	32.77 ± 16.73	45.97 ± 23.14	5.014	< 0.05		94.96 ± 85.96	33.38 ± 36.85	4.105	< 0.05
S7	34.42 ± 27.18	40.79 ± 22.09	2.521	< 0.05		100.95 ± 86.57	32.86 ± 30.23	4.83	< 0.05
S8	30.44 ± 26.95	44.75 ± 39.61	4.877	< 0.05		105.97 ± 94.58	33.87 ± 33.44	4.493	< 0.05
S9	36.21 ± 29.37	45.13 ± 29.37	4.374	< 0.05		96.92 ± 86.47	34.78 ± 37.62	4.155	< 0.05
S10	37.10 ± 27.54	47.98 ± 29.08	4.647	< 0.05		104.39 ± 90.32	34.46 ± 35.53	4.399	< 0.05
S11	40.92 ± 32.34	49.84 ± 32.27	3.390	< 0.05		98.00 ± 88.76	35.03 ± 34.21	4.241	< 0.05
S12	40.24 ± 22.70	45.43 ± 21.20	2.704	< 0.05		97.53 ± 85.58	30.49 ± 32.78	4.845	< 0.05
S13	28.45 ± 18.92	38.97 ± 21.52	4.845	< 0.05		102.41 ± 91.98	33.74 ± 33.13	4.522	< 0.05
S14	31.75 ± 22.24	40.39 ± 24.62	5.046	< 0.05		82.85 ± 80.32	24.64 ± 27.49	4.371	< 0.05
S15	33.68 ± 21.04	43.25 ± 24.02	3.081	< 0.05		107.57 ± 95.83	29.81 ± 29.20	4.866	< 0.05
S16	36.33 ± 22.36	44.71 ± 25.96	2.956	< 0.05		92.44 ± 78.39	27.39 ± 24.56	5.039	< 0.05
WM	36.38 ± 19.55	46.03 ± 18.65	5.789	< 0.05		99.93 ± 81.42	33.29 ± 30.89	4.644	< 0.05
MDEA	47.39 ± 41.22	59.77 ± 44.08	6.206	< 0.05					
BG	3.14 ± 2.48	6.17 ± 4.03	6.052	< 0.05					

Note: SD, standard deviation; SNR, signal-to-noise ratio; WM, whole myocardium; MDEA, myocardium delayed enhancement area; BG, background

### P_area_ assessment

The myocardial enhancement area was semi-quantitatively analyzed using various SD thresholds and the FWHM method. For the 2SD (Fig. 3b.**I, Supplementary Figs. 1**), 3SD (Figs. 2 and 3b.II, Supplementary Fig. 1), and 5D methods (Figs. 2 and 3b.IV, Supplementary Fig. 1), the P_area−DL_ values for the overall myocardium were higher than the corresponding P_area−O_ values for all 16 segments. For the 4SD method, the P_area−DL_ values of the overall myocardium were higher than the corresponding P_area−O_ values only in S1–S12 (Figs. 2 and 3b.III, Supplementary Fig. 1). For the FWHM method (Figs. 2 and 3.c, Supplementary Fig. 1), the P_area−DL_ values were slightly higher than the corresponding P_area−O_ values for all segments.

Regarding the DLR-based P_area_, the overall different threshold and FWHM-based P_area−DL_ values were higher than those based on any other approach (all *P* < 0.05). Regarding the P_area−O_, the values for the 2SD threshold were significantly higher than those based on other approaches (all *P* < 0.05) (Table 2).

**Table 2:** Differences between different-threshold and FWHM methods

	LGE_DL_				LGE_O_		
		t	*p*			t	*p*
2SD P_area_ vs. 3SD P_area_	41.32 ± 12.78 vs. 39.83 ± 16.58	1.454	>0.05		32.81 ± 12.59 vs. 31.41 ± 16.07	1.808	>0.05
2SD P_area_ vs. 4SD P_area_	41.32 ± 12.78 vs. 30.57 ± 15.25	8.390	< 0.05		32.81 ± 12.59 vs. 23.96 ± 12.79	7.644	< 0.05
2SD P_area_ vs. 5SD P_area_	41.32 ± 12.78 vs. 28.53 ± 12.92	12.072	< 0.05		32.81 ± 12.59 vs. 19.98 ± 12.73	11.836	< 0.05
2SD P_area_ vs. FWHM P_area_	41.32 ± 12.78 vs. 17.25 ± 11.22	10.567	< 0.05		32.81 ± 12.59 vs. 17.18 ± 11.12	7.375	< 0.05
3SD P_area_ vs. 4SD P_area_	39.83 ± 16.58 vs. 30.57 ± 15.25	10.342	< 0.05		31.41 ± 16.07 vs. 23.96 ± 12.79	7.468	< 0.05
3SD P_area_ vs. 5SD P_area_	39.83 ± 16.58 vs. 28.53 ± 12.92	10.963	< 0.05		31.41 ± 16.07 vs. 19.98 ± 12.73	14.059	< 0.05
3SD P_area_ vs. FWHM P_area_	39.83 ± 16.58 vs. 17.25 ± 11.22	8.878	< 0.05		31.41 ± 16.07 vs. 17.18 ± 11.12	5.695	< 0.05
4SD P_area_ vs. 5SD P_area_	30.57 ± 15.25 vs. 28.53 ± 12.92	2.142	< 0.05		23.96 ± 12.79 vs. 19.98 ± 12.73	5.474	< 0.05
4SD P_area_ vs. FWHM P_area_	30.57 ± 15.25 vs. 17.25 ± 11.22	5.733	< 0.05		23.96 ± 12.79 vs. 17.18 ± 11.12	3.048	< 0.05
5SD P_area_ vs. FWHM P_area_	28.53 ± 12.92 vs. 17.25 ± 11.22	4.964	< 0.05		19.98 ± 12.73 vs. 17.18 ± 11.12	1.314	>0.05

Note: LGE_DL_, deep learning-based reconstruction late gadolinium enhancement; LGE_O_, conventionally constructed late gadolinium enhancement; P_area_, percentage of myocardial enhancement area; FWHM, full width at half maximum

### Assessment of the consistency of the quantitative measurements

**Fig. 4:**
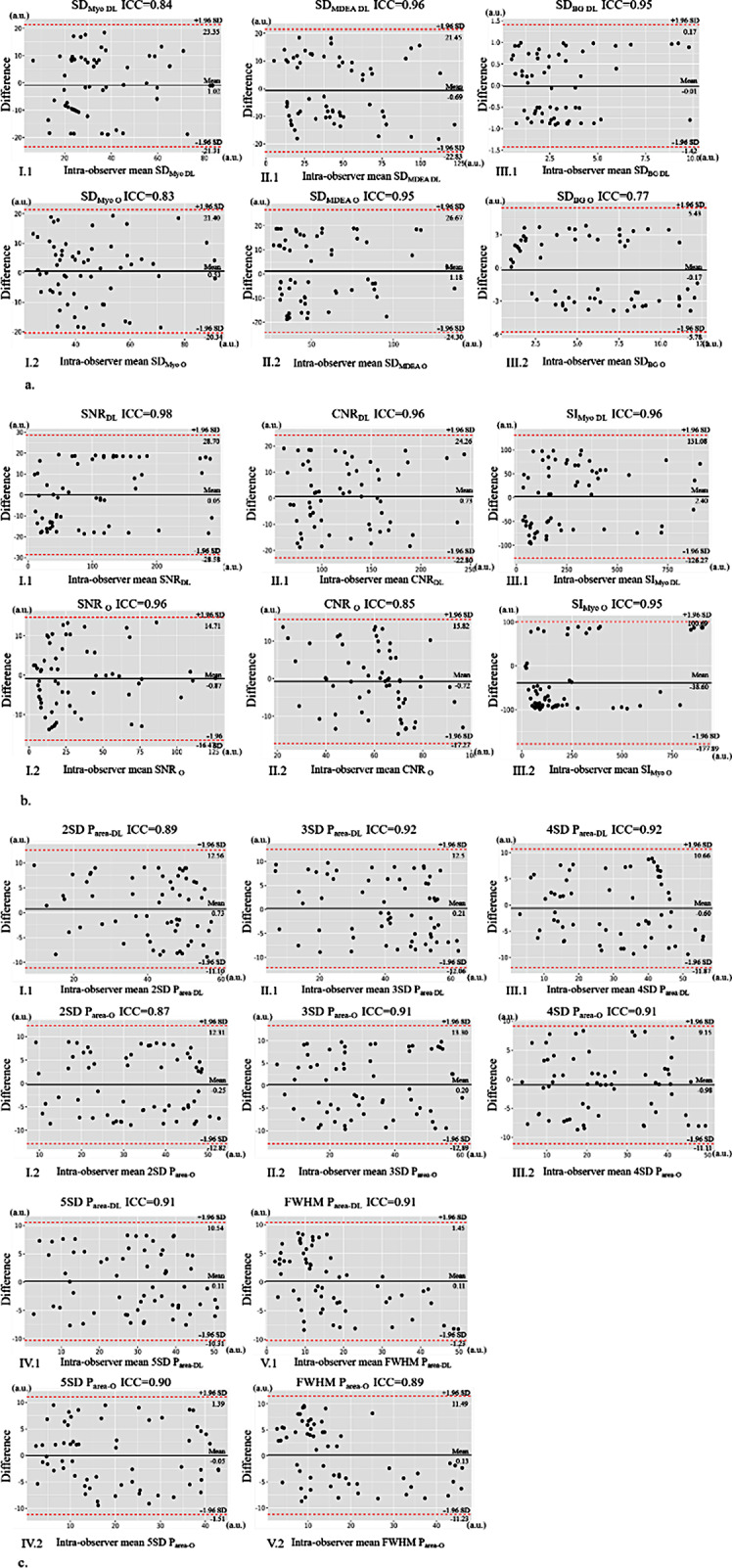
Bland–Altman plots for the intra-observer (a) SD of myocardium, enhancement area, and background noise; (b) SNR, CNR, SIMyo; (c) 2SD, 3SD, 4SD, 5SD, FWHM; 95% confidence intervals are labelled. There is a very good interstudy agreement for SD and FWHM methods. (1) LGE_DL_ images, (2) LGE_O_ images. Note: DL, deep learning late gadolinium enhancement; O: original late gadolinium enhancement; SD_Myo_: standard deviation of normal myocardium; SD_MDEA_: standard deviation of myocardial delayed enhanced area; SD_BG_: standard deviation of noise at the corner (background) of images; SNR: signal-to-noise ratio; CNR: contrast-to-noise ratio; SI_Myo_: signal intensity of normal myocardium; P_area_: percentage of myocardial enhancement area; 2, 3, 4, and 5SD threshold methods: mean P_area_ respectively adding 2, 3, 4, and 5 times of standard deviation of P_area_ as the threshold for myocardial enhancement area; FWHM: full width at half maximum; LGE_DL_: deep learning-based reconstruction late gadolinium enhancement; LGE_O_: conventionally constructed late gadolinium enhancement

**Fig. 5:**
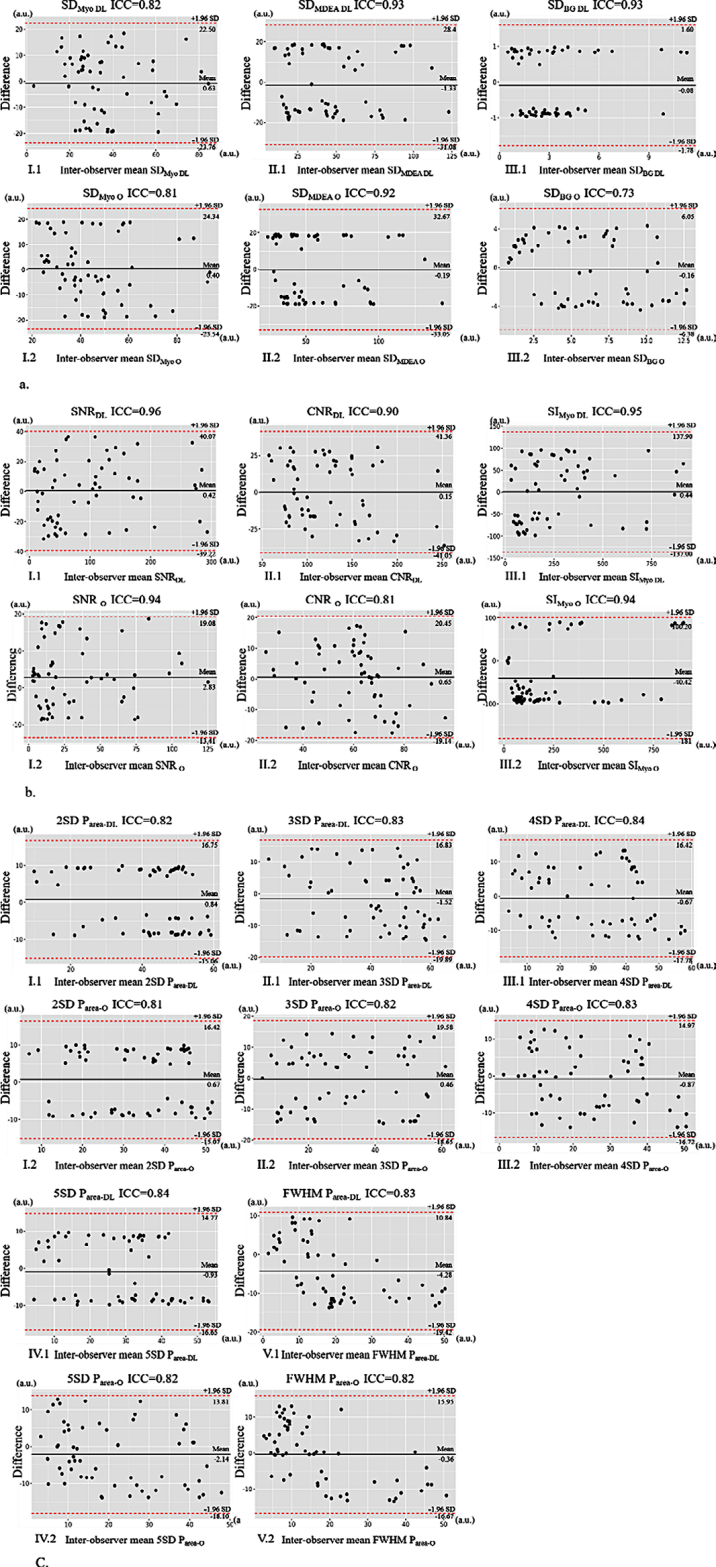
Bland–Altman plots for the inter- observer analysis; (a) SD of myocardium, enhancement area, and background noise; (b) SNR, CNR, SIMyo; (c) 2SD, 3SD, 4SD, 5SD, FWHM; 95% confidence intervals are labelled. There is a very good interstudy agreement for SD and FWHM methods. (1) LGE_DL_ images, (2) LGE_O_ images. Note: DL: deep learning late gadolinium enhancement; O: original late gadolinium enhancement; SD_Myo_: standard deviation of normal myocardium; SD_MDEA_: standard deviation of myocardial delayed enhanced area; SD_BG_: standard deviation of noise at the corner (background) of images; SNR: signal-to-noise ratio; CNR: contrast-to-noise ratio; SI_Myo_: signal intensity of normal myocardium; P_area_: percentage of myocardial enhancement area; 2, 3, 4, and 5SD threshold methods: mean P_area_ respectively adding 2, 3, 4, and 5 times of standard deviation of P_area_ as the threshold for myocardial enhancement area; FWHM: full width at half maximum; LGE_DL_: deep learning-based reconstruction late gadolinium enhancement; LGE_O_: conventionally constructed late gadolinium enhancement

The degree of intra- and inter-observer agreement for the objective measurements (SD_Myo_, SD_MDEA_, SD_BG_, SNR, CNR, and SI_Myo_) and P_area_ between LGE_DL_ and LGE_O_ images was good based on the various SD and FWHM methods (for objective measurements: all ICCs > 0.60, all *P* < 0.05; for P_area_: all ICCs > 0.70, *P* < 0.05). These measurements were better for LGE_DL_ images than for LGE_O_ images (Figs. 4 and 5).

### Analysis and comparison of diagnostic efficacy

**Fig. 6:**
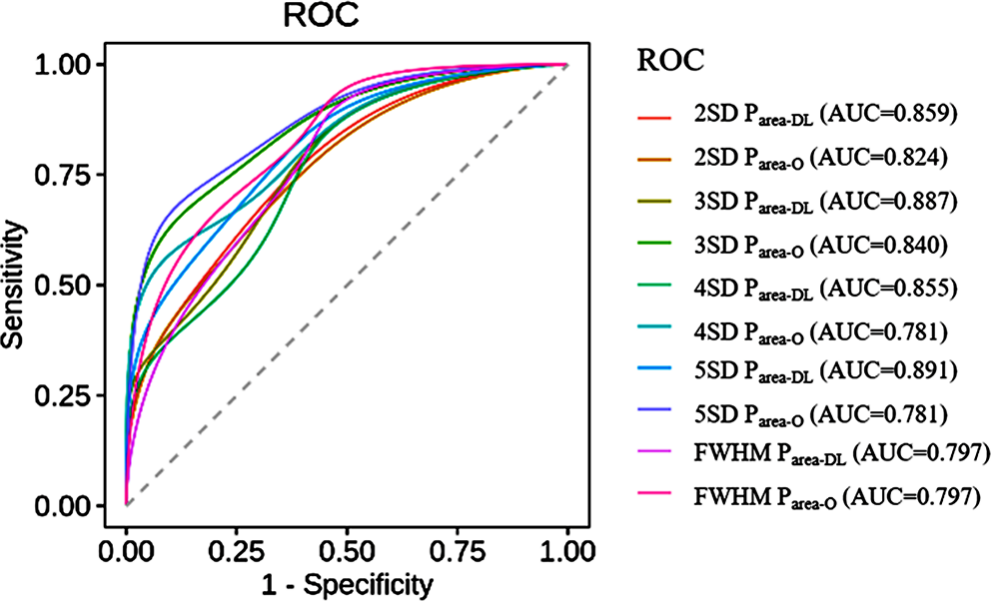
Diagnostic efficacy for UMI. Note: UMI: unrecognised myocardial infarction; SD: standard deviation; P_area-DL_, percentage of myocardial enhancement area with deep learning late gadolinium enhancement; P_area-O_, percentage of myocardial enhancement area with original late gadolinium enhancement; 2, 3, 4, and 5SD threshold methods, mean P_area_ respectively adding 2, 3, 4, and 5 times of standard deviation of P_area_ as the threshold for myocardial enhancement area; FWHM: full width at half maximum

All SD methods exhibited good diagnostic efficacy for UMI, with AUC values of the ROC curves ≥ 0.78. The P_area−DL_ based on the 5SD threshold method exhibited the optimal diagnostic efficacy of 0.891 (sensitivity = 0.688 and specificity = 1). For the conventional imaging enhancement, the P_area−O_ based on the 3SD method exhibited the optimal diagnostic efficacy of 0.840. The diagnostic efficacy was better for LGE_DL_ images than for LGE_O_ images for UMI detection for every SD threshold method, whereas it was not different between LGE_DL_ and LGE_O_ parameters based on the FWHM method (Table 3; Fig. 6).

**Table 3:** Area under the curve (AUC) for differentiation of UMI or non-UMI groups

*P*_area_	Area	Standard error	*P*	Approaching 95% confidence interval	
				Lower limit	Upper limit
2SD P_area−DL_	0.859	0.066	< 0.05	0.730	0.988
2SD P_area−O_	0.824	0.073	< 0.05	0.681	0.967
3SD P_area−DL_	0.887	0.057	< 0.05	0.775	0.998
3SD P_area−O_	0.840	0.069	< 0.05	0.705	0.975
4SD P_area−DL_	0.855	0.066	< 0.05	0.725	0.986
4SD P_area−O_	0.781	0.084	< 0.05	0.616	0.947
5SD P_area−DL_	0.891	0.056	< 0.05	0.781	0.999
5SD P_area−O_	0.781	0.085	< 0.05	0.615	0.947
FWHM P_area−DL_	0.797	0.079	< 0.05	0.642	0.951
FWHM P_area−O_	0.797	0.079	< 0.05	0.643	0.951

Note: SD, standard deviation; P_area−DL_, percentage of myocardial enhancement area with deep learning late gadolinium enhancement; P_area−O_, percentage of myocardial enhancement area with original late gadolinium enhancement; 2, 3, 4, and 5SD threshold methods, mean P_area_ respectively adding 2, 3, 4, and 5 times of standard deviation of P_area_ as the threshold for myocardial enhancement area; FWHM, full width at half maximum

## Discussion

This study compared LGE_O_ and LGE_DL_ images based on different SD thresholds and the FWHM method. The significant differences in P_area_ values between LGE_O_ and LGE_DL_ images for the SD threshold methods but not for the FWHM method suggested that the STRM should be ≥ 3, regardless of whether conventional or DLR-based LGE images are used, as previously reported. An STRM ≥ 4 and P_area−DL_ values based on the 5SD threshold exhibited the highest diagnostic efficacy for detecting UMI. Additionally, the LGE_DL_ images generated in this study could display the delayed enhancement area in patients with UMI for the first time, with significantly better image quality than was previously achievable with LGE_O_ images, such as artifacts in the myocardium, intensified foci and lower background noise, lower SD, and higher SNR and CNR values in all patients with UMI. Thus, LGE_DL_ imaging can improve diagnostic confidence without impacting diagnostic efficacy.

The presence of an infarction in patients with UMI is a critical feature for predicting adverse cardiac events [26–28]. The P_area_ on LGE images is the most frequently used direct indicator of irreversible damage at the pathological tissue level and can predict the treatment response to cardioprotective interventions [29, 30]. However, the clinical approach for quantifying the myocardial enhancement area is not uniform, with SD thresholds used in some instances and the FWHM method employed in others. Additionally, the generation of LGE images using conventional reconstruction and DLR-based methods is inconsistent. Generally, an STRM ≥ 3SD is the optimal reference threshold for clinical use. Quantifying the SD thresholds depends predominantly on the SI and SD of the ROIs drawn in the distal normal myocardium; however, the image quality of the remote normal myocardium may affect the visual sketching of the area to avoid the delayed lesion intensification on LGE_DL_ images [31]. For example, using a lower SD threshold of the distal myocardium leads to a significantly lower threshold for encompassing the extent of delayed enhancement, resulting in underestimation [13]. The SD values, including SD_Myo_, SD_MDEA_, and SD_BG_ of the LGE_DL_ images, showed similar patterns and were smaller than those of the LGE_O_ images, consistent with a previous DLR liver study [25]. Higher SNR and CNR values on LGE_DL_ images than on LGE_O_ images corresponded to improved inter- and intra-reader consistency of P_area_ measurements, indicating a more precise outline of the endocardium, epicardium, and foci boundary in the LGD_DL_ images because of the lower noise levels and fewer motion artifacts, especially in S1 and S16. DL plays a pivotal role in the field of medical image segmentation [17–21]. Currently, manual delineation is subject to certain variabilities. In the future, integrating artificial intelligence-based automatic segmentation optimization may reduce the inconsistencies associated with manual delineation [22, 25–28]. The incremental change in P_area_ values was inconsistent between segments; for example, S12, a middle segment of the lateral wall, exhibited a higher P_area_ on LGE_DL_ images than on LGE_O_ images, possibly due to less interference from artifacts and clearer edges of the lesion. Regarding the SD methods, the 4SD and 3SD threshold approaches in this study resulted in the highest inter- and intra-reader consistency for P_area−DL_ and the highest intra-reader consistency for P_area−O_. Therefore, threshold selection for image reconstruction based on conventional and DL-based approaches should be considered cautiously. Consistent with previous findings [12], the P_area−DL_ did not statistically differ from the P_area−O_ values when the FWHM method was used, as the technique only results in noise reduction without altering information fidelity on LGE_DL_ images. It yields highly reproducible and consistent enhanced areas regardless of the underlying etiologies for assessing the severity and extent of MI and other myocardial diseases [13, 16, 26, 32, 33].

This was the first study to evaluate and directly compare LGE_DL_ and LGE_O_ images of delayed intensification foci in patients with UMI. The diagnostic performance of the P_area−DL_ was higher than that of the P_area−O_ for the threshold approaches, especially for the P_area−DL_ based on the 5SD threshold, which exhibited the best AUC (0.891). For LGE_O_ images, the P_area−O_ based on the 3SD threshold exhibited the optimal AUC of 0.840, consistent with data from previous studies recommending using an STRM ≥ 3SD for infarct size. This study recruited patients with UMI without clinically significant cardiogenic chest pain and with a relatively small range of reinforcing foci; these results confirm that the 3SD threshold is sufficient for conventional LGE images. In contrast, a threshold ≥ 4SD should be used for DLR LGE images to optimize the intra- and inter-reader agreement and diagnostic efficacy. The diagnosis of the extent of infarction in UMI-related cases using the 4SD threshold was possibly a more reliable parameter for LGE_O_ and LGE_DL_ images despite the better diagnostic efficacy of the 5SD threshold for LGE_DL_ imaging. Furthermore, the detection rate of UMI was 67% (63/91); this rate was similar for LGE_O_ and LGE_DL_ images despite the better image quality and more reliable assessment of pathological features on LGE_DL_ imaging.

This study has some limitations. First, all participants were recruited using a single-center design, and only those who underwent an MR examination were included for analysis, limiting the generalizability of our results. Despite LGE images with high diagnostic accuracy of MI detection, the final diagnosis relies on experienced radiologists due to the lack of pathological validation for delayed enhancement areas on LGE images. Therefore, to enhance the robustness of result generalization, multicenter and large data, including comparison of P_area−DL_ and P_area−O_ using various SD and FWHM methods and validation of the accuracy and reliability for UMI diagnosis should be considered for future LGE_O_ or LGE_DL_.

## Conclusions

The selection of SD thresholds for LGE_DL_ (≥ 4SD) and LGEO (≥ 3SD) images was recommended for future research, as the difference between P_area−DL_ and P_area−O_ affected diagnostic efficacy and clinical decision-making in patients with UMI. Moreover, P_area−DL_ and P_area−O_ were similar when the FWHM method was used, implying LGE_DL_ images retained informational integrity. Despite the same UMI detection rates between LGE_O_ and LGE_DL_ images, the LGE_DL_ images showed superior image quality and reliable features for diagnosis with more confidence. Therefore, STRM selection and diagnostic outcomes should be carefully utilized and interpreted, particularly for DLR-based CMR images.

### Electronic supplementary material

Below is the link to the electronic supplementary material.

Supplementary Material 1

## Data Availability

The datasets generated or analyzed during the study are available from the corresponding author on reasonable request.

## Abbreviations

MI: Myocardial infarction
UMI: Unrecognized myocardial infarction
DLR: Deep learning reconstruction
SNR: Signal-to-noise ratio
CNR: Contrast-to-noise ratio
P_area_: Percentage of enhanced area
STRM: Signal threshold versus reference mean
SD: Standard deviation
FWHM: Full width at half maximum
CMR: Cardiac magnetic resonance
LGE_O_: Conventionally constructed late gadolinium enhancement
LGE_DL_: Deep learning-based reconstruction late gadolinium enhancement
MRI: Magnetic resonance imaging
DL: Deep learning
cTn: Cardiac troponin
ROI: Region of interest
SI: Signal intensity
ICC: Intraclass correlation coefficients
ROC: Receiver operating characteristic
AUC: Area under the curve
