# Correction: Evaluation of deep learning-based reconstruction late gadolinium enhancement images for identifying patients with clinically unrecognized myocardial infarction

**DOI:** 10.1186/s12880-024-01375-5

**Published:** 2024-07-24

**Authors:** Xuefang Lu, Weiyin Vivian Liu, Yuchen Yan, Wenbing Yang, Changsheng Liu, Wei Gong, Guangnan Quan, Jiawei Jiang, Lei Yuan, Yunfei Zha

**Affiliations:** 1 Department of Radiology, Renmin Hospital of Wuhan University, No. 238 Jiefang Road, Wuchang District, Wuhan, 430060 China; 2 MR Research, GE Healthcare, Beijing, China; 3 GE Healthcare, Beijing, China; 4 Computer School, Wuhan University, Wuhan, China; 5 Information Center, Renmin Hospital of Wuhan University, Wuhan, China

**Correction to: BMC Medical Imaging (2024) 24:127**

10.1186/s12880-024-01308-2

Following the publication of the Original Article, the authors reported an error in the section of CMR examination and image construction.

**Incorrect**:

Field of view = 34 mm.

**Correct**:

Field of view = 34 cm × 34 cm.

The Original Article has been corrected.

