# Supplementary material for: Evaluation of deep learning-based reconstruction late gadolinium enhancement images for identifying patients with clinically unrecognized myocardial infarction
Source: BMC Med Imaging. 2024 May 31;24:127. doi: 10.1186/s12880-024-01308-2 (PMC11141010; doi:10.1186/s12880-024-01308-2)
Supplement: Supplementary file 1 — Supplementary Material 1 [file 12880_2024_1308_MOESM1_ESM.docx]

**Additional files**


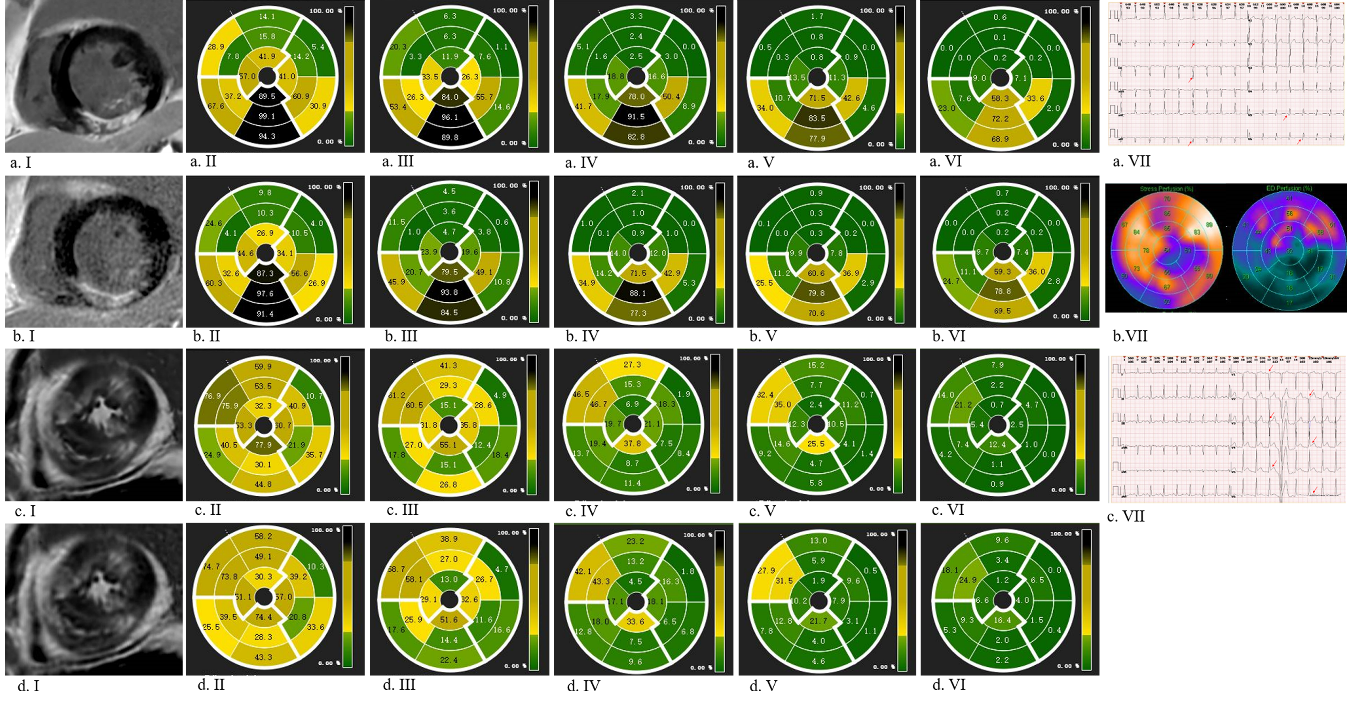


**Supplementary Fig.1.** Schematic diagram of P_area_ using accordingly (II) 2SD, (III) 3SD, (IV) 4SD, (V) 5SD, and (VI) FWHM methods for (a, c) LGE_DL_ images, (b, d) LGE_O_ images, and (a. VII, c. VII) electrocardiogram (ECG) of a UMI patient and a non-UMI patient. (a, c) shows clearer, less noisy, more uniform normal myocardial signal and better contrast between enhancement area and normal myocardium than (b, d). The UMI patient underwent stress perfusion myocardium and received an intravenous injection of 20 mCi 99mTc-MIBI. (b. VII) The stress perfusion maps as support P_area_ maps with clearer myocardium enhancement in the enlarged left ventricle, with morphological anomaly, relatively light sparsity of 20 mCi 99mTc-MIBI (a radiation tracker, RT) in the middle and basal segments of anterior wall and the middle segment of the anterioseptal wall, relatively strong sparsity of RTs in the apex, the apical segment of the septal wall, the middle and basal segments of the posteroseptal wall, the apical, middle, and basal segments of the inferior wall, the apical segment of the lateral wall, and the middle and basal segments of posterolateral, and normal perfusion in the remaining myocardium.

*Note: SD: standard deviation; 2, 3, 4, and 5SD threshold methods: mean P_area_ respectively adding 2, 3, 4, and 5 times of standard deviation of P_area_ as the threshold for myocardial enhancement area; FWHM: full width at half maximum; UMI: unrecognized myocardial infarction.*
